# Intelligence Architectures and Machine Learning Applications in Contemporary Spine Care

**DOI:** 10.3390/bioengineering12090967

**Published:** 2025-09-09

**Authors:** Rahul Kumar, Conor Dougherty, Kyle Sporn, Akshay Khanna, Puja Ravi, Pranay Prabhakar, Nasif Zaman

**Affiliations:** 1 Department of Biochemistry and Molecular Biology, University of Miami Miller School of Medicine, 1600 NW 10th Ave, Miami, FL 33136, USA; rxk641@miami.edu; 2 Sidney Kimmel Medical College, Thomas Jefferson University, 1025 Walnut St., Philadelphia, PA 19107, USA; cjd127@students.jefferson.edu (C.D.); aya156@students.jefferson.edu (A.K.); 3 Norton College of Medicine, Upstate Medical University, Syracuse, NY 13210, USA; spornk@upstate.edu; 4 Department of Biology, University of Michigan, 500 S State St., Ann Arbor, MI 48109, USA; puja.mi.us@gmail.com; 5 Albany Medical College, 43 New Scotland Ave, Albany, NY 12208, USA; prabhap@amc.edu; 6 Human-Machine Perception Laboratory, Department of Computer Science, University of Nevada Reno, 1664 N. Virginia St. LME 314, Reno, NV 89557, USA

**Keywords:** spine surgery, artificial intelligence, machine learning, predictive modeling, neural networks, spinal diagnostics, computer vision, surgical robotics, clinical decision support, biomedical informatics, musculoskeletal imaging, outcome prediction, precision medicine

## Abstract

The rapid evolution of artificial intelligence (AI) and machine learning (ML) technologies has initiated a paradigm shift in contemporary spine care. This narrative review synthesizes advances across imaging-based diagnostics, surgical planning, genomic risk stratification, and post-operative outcome prediction. We critically assess high-performing AI tools, such as convolutional neural networks for vertebral fracture detection, robotic guidance platforms like Mazor X and ExcelsiusGPS, and deep learning-based morphometric analysis systems. In parallel, we examine the emergence of ambient clinical intelligence and precision pharmacogenomics as enablers of personalized spine care. Notably, genome-wide association studies (GWAS) and polygenic risk scores are enabling a shift from reactive to predictive management models in spine surgery. We also highlight multi-omics platforms and federated learning frameworks that support integrative, privacy-preserving analytics at scale. Despite these advances, challenges remain—including algorithmic opacity, regulatory fragmentation, data heterogeneity, and limited generalizability across populations and clinical settings. Through a multidimensional lens, this review outlines not only current capabilities but also future directions to ensure safe, equitable, and high-fidelity AI deployment in spine care delivery.

## 1. Introduction

In this comprehensive narrative review, we analyze how artificial intelligence (AI) and machine learning (ML) technologies are shaping contemporary spine surgery and spine care. By examining advanced applications from diagnostic imaging to predictive genomics, we take an in-depth look at how sophisticated AI algorithms are revolutionizing multiple domains of orthopedic and spine-focused medicine, including automated radiological interpretation, surgical planning optimization, robotic-assisted procedures, and personalized risk stratification through genomic analysis. Current evidence demonstrates that AI-powered systems such as Aidoc’s cervical spine fracture detection [1], Zebra Medical Vision’s vertebral compression fracture identification [2], and SpineNet’s comprehensive spinal pathology analysis [3], among other systems and software, achieve diagnostic accuracies comparable to or exceeding specialist radiologists. In essence, we strongly believe that at the very least, these tools can be used alongside clinician practice as a secondary guide. Furthermore, emerging clinical decision support platforms, including Suki AI, Nuance Dragon Ambient eXperience (DAX), and Ambience Healthcare, are streamlining documentation workflows and enhancing physician-patient interactions through ambient intelligence capabilities [4]. In a similar vein, as many robotic systems like the Mazor X Stealth Edition, ExcelsiusGPS, and ROSA Spine with AI-driven surgical planning are being increasingly tested and even taught to incoming residents [5], we believe there is an ongoing shift toward precision-guided interventions that minimize invasive approaches while maximizing surgical accuracy. Genomics is also finding its role in risk prediction and stratification, as genome-wide association studies (GWAS) and deep learning models are enabling new insights into genetic predispositions for pathologies and outcomes alike, thus allowing clinicians to now factor in patient-specific risk profiles [6]. In this review, we synthesize current evidence across these diverse applications while addressing implementation challenges, regulatory considerations, and future directions for AI integration in spine care.

## 2. Methodology

This narrative review was conducted as a structured narrative synthesis of peer-reviewed literature, regulatory reports, and industry documentation related to artificial intelligence (AI) and machine learning (ML) applications in spine care. The aim was to provide a comprehensive overview of validated AI tools, clinical decision support systems, genomic integration models, and robotic surgical platforms in contemporary spinal diagnostics and intervention; it does not present original experimental data.

### 2.1. Literature Search Strategy

The literature search was conducted between January 2024 and May 2025 across PubMed, Scopus, IEEE Xplore, and Google Scholar. Search terms included “artificial intelligence AND spine surgery,” “machine learning AND spinal imaging,” “robotic spine systems,” “spinal genomics,” “polygenic risk score AND spine,” “ambient clinical intelligence,” and “federated learning in healthcare.” Boolean operators were used to refine the scope. References were also drawn from FDA medical device databases and reports (e.g., HealthVCF 510(k) clearance, 2020 [7,8]), corporate white papers, and open-access AI repositories.

Only English-language sources published between 2007 and 2025 were considered. Priority was given to high-impact clinical studies, FDA-approved systems, externally validated models, and articles published in peer-reviewed journals with known impact factors. Foundational studies on algorithm development and AI architectures dating back to 2007 (e.g., Gstoettner et al. on Cobb angle measurement accuracy [9]) were included to provide historical context.

### 2.2. Inclusion and Exclusion Criteria

Studies were included if they met at least one of the following criteria:

- Described AI/ML systems used in spine diagnostics, surgical planning, or outcome prediction (e.g., SpineNetV2 2017 [3,10,11,12], Aidoc Cervical Spine AI Version 1, 2021 [1,13,14,15], ExcelsiusGPS, 2024 [5,16,17]).
- Reported external validation results of AI-based tools in spinal imaging (e.g., Yeh et al., 2021 [18]; Nigru et al., 2024 [3]).
- Detailed FDA-cleared robotic systems with spine-specific capabilities (e.g., Mazor X, 2021 [3,5,16,19], VELYS, 2024 [10,20]).
- Investigated genomic risk stratification or pharmacogenomic frameworks applied to spinal pathologies (e.g., Sigala et al., 2023 [6]; Salo et al., 2024 [21]).
- Described ambient clinical intelligence or documentation automation tools with demonstrated implementation in spine-related clinical settings (e.g., Nuance DAX, 2024 [22]; Ambience Healthcare, 2025 [23]).

Excluded were unpublished conference abstracts, non-peer-reviewed preprints (unless containing novel datasets or architectures), non-English publications, and studies lacking clear methodological description or validation metrics.

### 2.3. Data Extraction and Synthesis

Key information extracted included the following: system/tool name, validation methodology (e.g., peer-reviewed study, FDA clearance, or real-world data), clinical domain of application (diagnostic imaging, robotics, genomics, etc.), performance metrics (e.g., AUC, sensitivity/specificity, error rates), and year of publication. Data were synthesized into thematic domains including (1) diagnostic imaging and automated classification; (2) morphometric and functional analysis; (3) surgical robotics and navigation; (4) genomic and pharmacogenomic personalization; (5) documentation and decision support; and (6) cost-effectiveness and health systems modeling.

The extracted data were organized into summary tables (e.g., Table 1: AI application overview; Table 2: implementation barriers) and visualized across domains to highlight clinical utility, limitations, and translational gaps.

### 2.4. Risk of Bias and Validation Considerations

To assess generalizability and real-world relevance, we prioritized studies that included external validation cohorts (e.g., SpineNetV2, validated in 2022–2024), FDA-approved platforms (e.g., HealthVCF, 2020 [7,8]; Aidoc, 2021 [1,13,14,48]), or independent cross-institutional replication. Risk of bias was addressed by identifying overfitting to synthetic datasets, geographic underrepresentation (e.g., urban-biased cohorts in CTSpine1K [49,50]), and algorithmic opacity (e.g., black-box decision-making in sentiment analysis tools). Studies were cross-referenced with regulatory databases and critical evaluations (e.g., Clark et al., 2023 [20]; Chouffani El Fassi et al., 2024 [51]) to ensure proper documentation of clinical applicability.

## 3. Current Applications of AI in Imaging and Radiological Analysis

### 3.1. Automated Detection and Classification of Spinal Pathologies

AI, and more importantly, the resultant sophisticated deep learning algorithms, are increasingly capable of detecting and classifying complex pathological conditions, largely changing clinical practice. Convolutional neural networks (CNNs) are a class of deep learning models designed to process images by automatically learning which visual features matter for a task (for example, detecting lesions on an X-ray or classifying a scan). Rather than requiring manually engineered image descriptors, CNNs ingest large, labeled imaging datasets and build a hierarchy of spatial features: early processing emphasizes simple patterns (edges, textures), while deeper processing combines those elements into organ- or pathology-level representations. This hierarchical, data-driven feature learning is a practical reason CNNs have advanced diagnostic image analysis. [24]

In clinical practice CNNs are applied to both image-level tasks (e.g., classification such as “tumor present/absent”) and pixel-level tasks (e.g., segmentation or localization), and they can produce auxiliary outputs such as heatmaps that help indicate influential regions. Their performance depends strongly on the quality and quantity of annotated training data and on robust validation, but when well-trained, they achieve high sensitivity and specificity across many imaging tasks [25,26,52,53,54,55]. (See Appendix A for technical details.)

Convolutional neural networks (CNNs) are increasingly integrated into routine radiographic evaluation of the spine. They can automate the detection, localization, and characterization of fractures and other pathologies across modalities, thereby reducing missed injuries and accelerating diagnosis. Research studies across radiography, CT, and dual-energy X-ray absorptiometry (DXA) demonstrate the breadth and performance of modern CNN approaches. On plain thoracolumbar radiographs, a deep convolutional neural network achieved an accuracy of 86.0%, sensitivity of 84.7%, and specificity of 87.3% for vertebral fracture detection; a performance that was non-inferior to orthopedic and spine surgeons [29]. On CT, a multistage system combining U-Net segmentation with graph convolutional networks (U-GCN) for detection, localization, and AO classification of acute thoracolumbar vertebral body fractures demonstrated vertebra-level sensitivity of 95.23%, accuracy of 97.93%, and specificity of 98.35% [56]. In DXA-based vertebral fracture assessment, an ensemble of CNNs achieved an area under the curve (AUC) of 0.94 with a sensitivity of 87.4% and a specificity of 88.4% for vertebral fracture identification; importantly, fractures identified by the CNNs predicted future nonvertebral and hip fractures comparably to expert readers, underscoring prognostic as well as diagnostic utility [30]. Recent reviews confirm that these AI models achieve very high diagnostic accuracy, especially for vertebral fractures. In one 2024 meta-analysis of 40 studies, AI tools yielded AUROC ≈ 0.92 for osteoporotic vertebral fracture diagnosis (and AUROC ≈ 0.87 overall for vertebral compression fractures) [36]. These pooled results reinforce that CNNs routinely approach or exceed expert radiologist performance in spine imaging.

Cervical spine findings mirror these advances. A hybrid transfer-learning approach that combined Inception-ResNet-v2 with U-Net for CT-based cervical vertebra fracture detection reported an overall accuracy of 98.44% on a large test set, outperforming radiologist predictions [31]. Likewise, a U-Net–based segmentation pipeline detected 87.2% of cervical spine fractures with a low false-positive rate, supporting its deployment in trauma workflows where rapid, reliable triage is critical [32]. Similarly, CNNs have shown excellent accuracy on cervical spine X-rays. Liawrungrueang et al. (2024) trained a CNN on lateral C-spine radiographs and achieved 92.1% overall accuracy for fracture detection (sensitivity ≈ 88.6%, specificity ≈ 95.7%), demonstrating that deep learning can reliably flag cervical fractures even on standard X-ray films [57]. Together, these modality-spanning results show that CNNs can match or exceed clinician performance for many spine-imaging tasks, offering standardized, fast screening and level-by-level characterization that augment radiologist interpretation and improve the timeliness of care. While some studies highlight limitations in generalizability, scan mode sensitivity, or reduced performance in certain clinical scenarios, the overall body of evidence supports the diagnostic utility of CNNs for this application [35,58,59,60]. Multiple systematic reviews and meta-analyses, as well as large cohort studies, consistently report that CNNs achieve high accuracy, sensitivity, and specificity for vertebral fracture detection on radiographs and related imaging modalities, often matching or approaching expert clinician performance [30,33,34,36,61]. 

Given their effectiveness, there has been a push for creation of commercial CNNs aimed at being implemented in hospital workflow, such as the Zebra Medical Vision’s FDA-cleared HealthVCF. HealthVCF version 5.1.1 is a CNN based software that accurately detects moderate-to-severe vertebral compression fractures (VCFs), which seamlessly integrates into the existing hospital Picture Archive and Communication (PAC) infrastructures [7], thus enabling the automatic transfer and analysis of CT scans without manual intervention [7]. In the detection of moderate-to-severe (Grade 2–3) VCFs, HealthVCF has demonstrated strong performance. A study focusing on incidental fracture detection using HealthVCF on chest and abdominal CT scans reported an overall diagnostic accuracy of 89.6% (95% CI: 87.4–91.5%) for moderate-to-severe VCFs [62]. This study detailed a sensitivity of 73.8%, a specificity of 92.7%, and an NPV of 94.8% [62].

Beyond its general accuracy, HealthVCF was able to identify fractures that radiologists failed to report in 42.8% of positive scans, and it identified 38 additional ones from the scans [62]. These findings highlight HealthVCF’s ability to achieve diagnostic performance non-inferior to human specialists and, sometimes, even detect fractures missed by human radiologists. Given these findings, AI can become a crucial safety net in clinical practice, helping to reduce human error, enhancing diagnostic completeness. Its integration into radiological workflow for fracture identification can help improve overall patient safety and quality of care. This augmentation of human capabilities is particularly valuable in high-volume settings where subtle findings might otherwise be overlooked.

In practice, these AI accuracies have been translated into clinical tools. For example, an FDA-cleared CNN triage system (Aidoc) for acute cervical spine fractures reported ~94.8% overall diagnostic accuracy (sensitivity ≈ 89.8%, specificity ≈ 95.3%) in one large study [63]. However, a subsequent real-world evaluation of the same Aidoc algorithm showed lower sensitivity (~54.9%) [63], highlighting that performance can vary across settings. Nonetheless, these results illustrate that modern AI platforms can achieve radiologist-level accuracy in spine fracture detection, though careful validation in diverse populations is needed.

Besides fractures, CNNs have been applied extensively in lumbar spine MRI to identify degenerative disk diseases. For example, herniated disks on axial T2 MRI can be detected and graded by deep learning models. Zhang et al. developed a two-stage deep model (Faster R-CNN for detection + ResNeXt101 for classification) to identify and grade lumbar disk herniation according to the Michigan State University (MSU) classification [64]. Their model achieved high detection accuracy (mean intersection over union ≈0.82 internally and 0.70 on an external set) and correctly classified herniation grade in about 87.7% of cases (internal test) [64]. In other words, the CNN could rapidly draw bounding boxes around disks and assign a herniation grade with “high consistency” to expert radiologists [64]. This illustrates a general workflow: a CNN first finds the disk (or region of interest) and then classifies its pathology. Since the MSU grading in this study was more objective than some older schemes, the model’s agreement with surgeons was high, indicating such systems could standardize interpretations and even potentially flag cases needing surgery.

CNNs can also grade disk degeneration (e.g., Pfirrmann grades). For instance, a recent YOLOv5-based model simultaneously detected and graded disk degeneration, herniation, and high-intensity zones on lumbar MRI [65]. It achieved precisions in the range of ~0.80–0.90 and recall ~0.84–0.94 for detecting disk herniations and degeneration (with Cohen’s kappa ~0.84 between the model and a senior surgeon) [65]. Such multi-task models integrate several classification steps into one network, improving efficiency. The study supports the notion that CNNs can automatically identify disk pathology on MRI (i.e., locating the affected disk and providing an objective severity grade) with performance rivaling human readers.

Lumbar spinal stenosis (LSS) is another area where CNNs have shown promise. Tumko et al. (2024) designed a three-stage CNN: one stage segments relevant anatomy, and two stages separately classify stenosis for central canal, lateral recess, and foraminal regions [66]. On an external test set of 150 MRI studies, their model’s detection of any stenosis and its grading (normal/mild/moderate/severe) was comparable to a panel of radiologists. In fact, the model’s sensitivity and specificity for detecting each subtype were very high, e.g., for central stenosis the CNN achieved sensitivity of 0.971 and area under the receiving operating chrematistics curve of 0.963, exceeding the average radiologist (0.786 and 0.842, respectively) [66]. This study suggests that CNNs can detect and classify LSS comparable to radiologists. Likewise, Hallinan et al. (2021) [37] trained a CNN to identify central canal, lateral recess, and foraminal stenosis in lumbar MRI. Their deep model’s agreement (κ ~0.92–0.96) with subspecialist radiologists was “almost perfect” for dichotomous classification of stenosis (normal/mild vs. moderate/severe). In other words, whether for assigning a grade or simply flagging significant stenosis, CNN-based tools can match the accuracy of experienced neuroradiologists in lumbar spine MRI [37]. These studies suggest that automated lumbar stenosis detection could improve efficiency and consistency in MRI reporting. AI has similarly excelled in other spine diagnostic tasks. For example, deep learning pipelines can estimate scoliosis Cobb angles with error ≲3.5° [67] matching human variability, and recent YOLOv8-based models for lumbar disk herniation detection achieved mAP ≈ 0.78 in validation sets [67]. These results confirm that AI tools now rival experts across a broad range of spine imaging diagnoses.

While some AI models are focused on detecting pathologies traditionally identified by radiologists, radiomics focuses on capturing patterns on radiographical images that are not visible to the human eye [38]. Radiomic models extract hundreds of features (e.g., intensity histograms, shape descriptors, texture matrices) from standard medical images (e.g., MRI, CT, X-ray). They then apply mathematical algorithms to quantify the spatial distribution of voxel intensities and their interrelationships, turning visual cues (e.g., differences in intensity, shape, texture) into numeric biomarkers [38]. In practice, a region of interest (e.g., a spinal disk or vertebra) is segmented by first-order (e.g., intensity), second-order (e.g., texture), and higher-order (e.g., wavelet or LoG-filtered) features [38]. These features are then compounded and fed into machine-learning models, augmenting standard radiology with objective indicators of subtle pathology.

The application of radiomics in spine relation pathologies ranges from grading spinal degeneration to determining ideologies of infectious spondylitis. For example, Xie et al. (2024) developed an MRI radiomics workflow to automatically classify cervical disk degeneration [68]. They extracted ~924 features from segmented disks on T1- and T2-weighted MRI and trained a random forest classifier. The combined T1–T2 model achieved a test AUC ≈ 0.95 for distinguishing low-grade from high-grade degeneration [68]. Crucially, higher-order texture features accounted for ~80% of the model’s predictive power [68], implying that subtle textural heterogeneity in the disk (imperceptible on routine MRI) drove the diagnosis. In other words, radiomics quantified microscopic variations in nucleus pulposus signal and annular appearance that are only qualitatively appreciated on standard images. This automated approach could standardize disk degeneration assessment, reducing dependence on subjective Pfirrmann grading.

Radiomics also enhances tumor characterization and prognostication in the spine. For instance, spinal multiple myeloma and metastatic lesions often look similar on MRI, but Cao et al. (2024) showed that a radiomics model using T2 and contrast-enhanced T1 MRI could distinguish myeloma from metastasis with a ~86% accuracy (AUC ≈ 0.87) [39]. In this study hundreds of texture and shape features were combined in a classifier, yielding good differential performance that would be difficult by eye alone. Beyond diagnosis, radiomics can predict treatment response; in spinal metastases treated with stereotactic radiotherapy, Chen et al. (2023) [41] found that radiomic features from pre-treatment MRI improved outcome prediction. Their combined radiomics–clinical model achieved AUC ≈ 0.83 for local control (versus AUC ≈ 0.73 using clinical factors alone). In other words, radiomic signatures on baseline MRI (e.g., reflecting tumor heterogeneity, edema, etc.) carried prognostic information inaccessible by conventional review. These examples illustrate that radiomics encodes latent biologic information in tumors (e.g., cell density, angiogenesis, necrosis) into quantifiable metrics, sharpening diagnostic and predictive accuracy in spine oncology.

Radiomics has likewise revealed hidden cues in non-neoplastic spine disorders. In infectious spondylitis, MRI radiomic signatures can differentiate etiologies that overlap with imaging. Qin et al. (2025) [69] extracted hundreds of radiomic features from spine MRIs in patients with tuberculous, brucellar, or pyogenic spondylitis and built a classifier model. The resulting nomogram achieved AUC ≈ 0.92 in training and ≈0.87 in testing, effectively distinguishing the three infection types. The authors concluded that their radiomics model could “gradually differentiate tuberculous spondylitis, brucellosis spondylitis, and pyogenic spondylitis”, a level of detail beyond routine MRI interpretation [69]. In a different domain, CT radiomics has been used to predict osteoporotic fracture risk. For example, Yang et al. (2025) analyzed postoperative spine CT scans of patients treated with vertebroplasty and found that a model combining CT-radiomic features with clinical factors predicted adjacent-level fracture (AUC ≈ 0.86) [40]. This suggests that radiomics can capture minute bone texture and density variations (microarchitecture deterioration) not apparent on standard images, flagging patients at high fracture risk.

Overall, these studies demonstrate that radiomics systematically uncovers image features (e.g., texture, shape, intensity patterns) that are usually imperceivable by the naked eye. By transforming subtle imaging heterogeneity into quantitative biomarkers, radiomics augments conventional spine imaging and enhances diagnostic precision in conditions ranging from degenerative disk disease to tumors, infections, and fractures. In essence, radiomics provides additional data beyond visual inspection, enabling earlier detection and more accurate characterization of spine pathology.

Despite their promise, current AI tools in spine imaging still require rigorous validation across diverse patient populations, careful integration into clinical workflows, and demonstration of consistent performance, as they are not yet universally equivalent to expert clinicians. Evidence from real-world evaluations highlights this concern. For example, researchers who processed approximately 10,000 CT scans with HealthVCF at a Danish hospital reported a sensitivity of 0.68 (68%, 95% CI 0.581–0.776) and a specificity of 0.91 (91%, 95% CI 0.89–0.928) [42]. The investigators noted that the algorithm’s performance was “poorer than expected” and concluded that the tested version was “not generalizable to the Danish population,” highlighting potential variability in real-world clinical settings [42]. While initial studies on HealthVCF have shown robust metrics, the observed lack of generalizability in a real-world setting underscores a critical challenge for all AI models: their consistent effectiveness across diverse patient populations and varied clinical environments.

The urgency of addressing this limitation is amplified by the fact that numerous AI systems entered clinical practice without undergoing thorough validation by the U.S. Food and Drug Administration (FDA) prior to 2025. Clark et al. examined 119 medical devices marketed as AI- or ML-enabled and found that 23 (19.3%) displayed discrepancies or ambiguities between their advertised capabilities and actual FDA clearance [20]. Moreover, many AI/ML tools undergo validation using synthetic or “phantom” datasets, such as Generative Adversarial Networks (GANs), which often fail to capture the complexity of real-world clinical conditions. Supporting this concern, Chouffani El Fassi et al. reported that 226 of 521 FDA-approved AI-enabled clinical devices lacked peer-reviewed validation and were not trained on genuine patient data [51]. These findings emphasize the critical need for strengthened regulatory oversight, stricter validation requirements, and mechanisms for continuous post-market monitoring. Without clinically representative training data and real-time performance auditing, so-called “shadow” AI systems risk perpetuating bias, jeopardizing patient safety, and obscuring liability.

### 3.2. Advanced Morphometric Analysis and Quantitative Assessment

AI-driven segmentation is rapidly transforming spinal radiographic imaging by automating the precise delineation and labeling of vertebral bodies, intervertebral disks, neural foramina, and other anatomical landmarks. Automated segmentation and labeling remove much of the repetitive manual workload, reduce inter-observer variability, and create the consistent regions of interest required for downstream quantification (e.g., Cobb angles, Pfirrmann grading, canal cross-sectional area). By converting anatomy into structured, pixel-accurate masks, these tools enable reproducible measurements, longitudinal monitoring, and the reliable extraction of radiomic features that feed diagnostic and prognostic models.

New tools, such as SpineNet by Jamaludin et al., represent a landmark achievement in comprehensive spinal analysis, as they can automate segmentation and label spinal structures across various imaging modalities (e.g., CT, MRI) [10,70]. This automated process allows for the one unified system to grade disk degeneration (e.g., Pfirrmann grades), endplate defects, bone marrow changes, foraminal stenosis, and spondylolisthesis [10,70]. Fortunately, many similar platforms are emerging, and external validation studies have confirmed that these platforms are beginning to show consistent performance across different institutional, socioeconomic, and patient settings (e.g., rural, urban, etc.) [9,11].

Other networks such as *Spine-GAN* have extended this approach by performing semantic segmentation of spinal anatomy: it segments intervertebral disks, vertebral bodies, and neural foramina on MRI to aid in diagnosing foraminal stenosis and degeneration [71]. More recent multi-stage pipelines combine diverse inputs: for instance, Windsor et al. (2024) describe an AI that first detects and labels vertebral bodies across T1, T2, and STIR MRI sequences and then applies Transformer-based networks to diagnose disk pathology, cord compression, metastases, and vertebral fractures [43]. Such end-to-end systems effectively replicate an expert reader by linking image registration and diagnosis in one workflow.

Critically, these advanced AI platforms are beginning to prove robust across settings. Open-source models like SpineNetV2, an updated second-generation version of SpinNet, have undergone external validation in independent datasets. In one study, SpineNetV2 showed “strong performance” in predicting disk pathologies: Cohen’s κ and related metrics exceeded 0.7 for most evaluated features, although foraminal stenosis and herniation were somewhat harder [3]. These results demonstrate that a model trained at one site can generalize with high reliability elsewhere. The availability of large, annotated datasets is accelerating progress: for example, the CTSpine1K resource contains over 11,000 labeled vertebrae (healthy and diseased), providing a rich training ground for segmentation algorithms [49]. When such algorithms are open-sourced or deployed via federated networks (e.g., TriNetX), researchers worldwide can contribute to and refine AI models, improving their performance across patient populations.

These AI automated segmentation advances are also opening new modality frontiers. Ultrasound spine images are notoriously difficult for analysis given their low contrast and speckled noise, but attention-based deep networks have made segmentation feasible. Jiang et al. (2022) introduced the ultrasound global guidance block network (UGBNet), which incorporates spatial and channel attention to segment vertebral landmarks in noisy ultrasound scans [72]. In their scoliosis study, UGBNet significantly outperformed baseline models, achieving a Dice segmentation score of 0.742 [72]. By focusing on long-range spatial context and feature channels, UGBNet can reliably trace bone contours that were previously hard to see. With such tools, clinicians can use portable ultrasound to visualize spine curvature in real time. In effect, attention-augmented U-Nets turn ultrasounds into more interpretable images, expanding AI spine analysis into new modalities.

**Figure 1:**
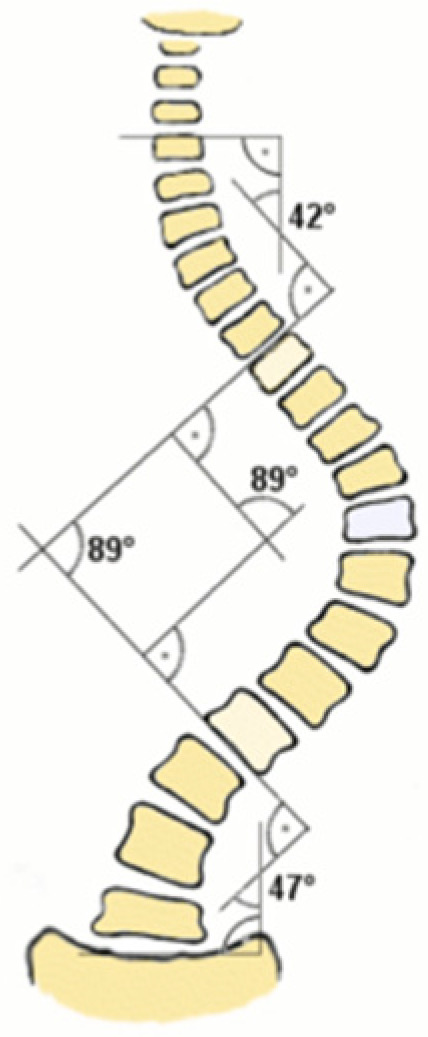
Cobb angle measurement in scoliosis. Permission is granted to copy, distribute and/or modify this document under the terms of the GNU Free Documentation License, Version 1.2 or any later version published by the Free Software Foundation, with no Invariant Sections, no Front-Cover Texts, and no Back-Cover Texts. This file is licensed under the Creative Commons Attribution-Share Alike 3.0 Unported license with permission from Wikimedia Commons [74].

**Figure 2:**
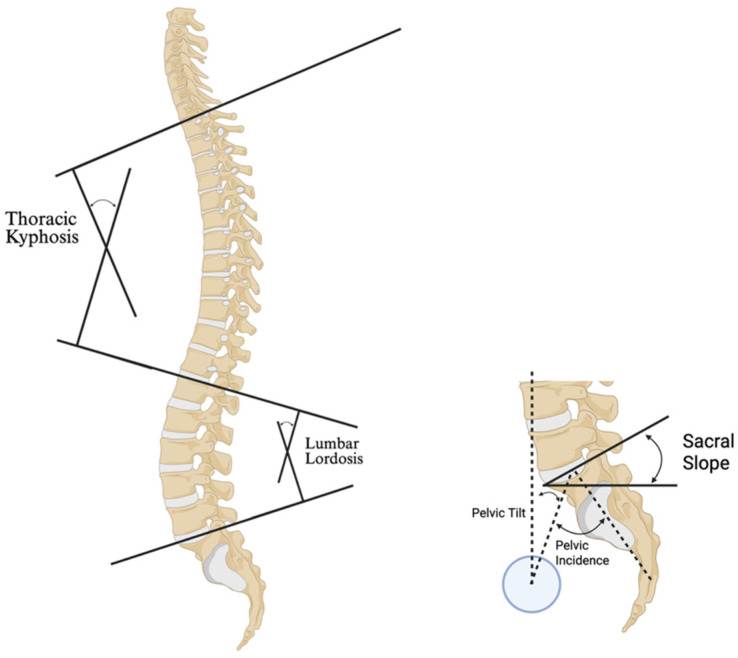
Graphical representation of spinal and pelvic alignment parameters that convolutional neural networks (CNNs) can automatically extract from whole-spine radiographs. Shown here are thoracic kyphosis, lumbar lordosis, sacral slope, pelvic tilt, and pelvic incidence. CNN models have also been demonstrated to measure additional parameters such as sagittal vertical alignment, coronal Cobb angles, and frontal pelvic asymmetry with accuracy comparable to human evaluators. Figure created with BioRender.com.

AI is similarly transforming quantitative measurements of spinal alignment. Conventional manual Cobb angle measurements have 95% confidence intervals on the order of 4–8°, while deep learning has markedly improved consistency with mean absolute errors consistently below 3° (Figure 1) [18]. Galbusera et al. demonstrated that a CNN can automatically measure lumbar lordosis, thoracic kyphosis, sagittal vertical alignment, coronal Cobb angle, and pelvic parameters (e.g., frontal pelvic asymmetry, sacral slope, pelvic tilt, and pelvic incidence) on whole-spine radiographs with accuracy parallel to human evaluators (Figure 2) [73]. In other words, AI can quickly replicate the full battery of clinical angles that a spine surgeon would manually compute, but in a fraction of the time and with less variability. Subsequent studies have extended these models to very large cohorts, showing that AI can process thousands of archived spine films to derive alignment statistics that would otherwise take months of manual work [44].

Deep networks have also been developed specifically for landmark localization. Yeh et al. (2021) trained models on 2,210 lateral spine X-rays of diverse pathology to detect 45 anatomical landmarks and automatically compute 18 key sagittal parameters (e.g., C2–C7 angle, SVA, pelvic tilt) [18]. Their ensemble model’s measurements were highly correlated with expert annotations; in fact, they matched clinician reliability in 15 of the 18 parameters [18]. This approach, first localizing landmarks, then calculating angles, means AI can handle even off-center or suboptimal films by focusing on consistent internal references. In practice, this yields near-expert performance on tasks like Cobb angle measurement, even if the X-ray is rotated or partially cut off.

These capabilities are being packaged into practical tools. For instance, CobbAngle Pro is a smartphone app that employs deep learning to analyze mobile photos of spine X-rays and output scoliosis curvature. Field users (e.g., combat medics or rural clinicians) can simply photograph the radiograph, and the app will identify vertebral endplates and draw Cobb angles automatically. Published reports indicate this app’s measurements agree closely with expert readers across mild, moderate, and severe scoliosis [45,46]. Since it is commercially available and designed for ease of use, CobbAngle Pro exemplifies how AI-driven morphometry is reaching frontline practice: it allows clinicians to perform reliable alignment analysis at the bedside or in austere settings, freeing them from tedious manual angle calculations [45,46].

Another advanced development is tissue segmentation. Models are now demonstrating automated quantification of paravertebral muscle characteristics, including cross-sectional areas and fatty infiltration patterns that correlate with functional outcomes and disability measures. A primary example of this is the CTSpine1K dataset, which has annotations for over 11,000 vertebrae including both healthy and pathological specimens [47]. The main benefit of CTSpine1k and related datasets, especially open-source datasets such as those available on TrinetX [47,50], is that they can objectively measure muscle quality and quantity on a large scale, thus enabling smaller research groups to study pathology and incidence without necessarily needed an IRB-dictated patient cohort.

Another significant application is AI-enabled image reconstruction and enhancement. Modern imaging modalities, such as MRI and CT, often face trade-offs between image resolution, signal-to-noise ratio, and scan time. AI tools, such as those developed by NVIDIA Clara Imaging, use deep learning to reconstruct clearer, higher-resolution images from lower-quality raw data [50]. This not only improves diagnostic confidence but also allows for faster scans, reducing patient discomfort and motion artifacts, which is particularly useful for spinal imaging where movement can compromise image quality.

Modern spine MRI and CT scans face inherent trade-offs between scan time, resolution, and noise. Recent deep learning (DL) methods have begun to overcome these limits by reconstructing high-quality images from undersampled or noisy data. In spine MRI, DL reconstruction can dramatically speed up exams while maintaining (or improving) image quality. For example, a prospective study of lumbar spine MRI found that a DL-reconstructed turbo spin-echo protocol (TSE-DL) cut scan time by ~45% (i.e., 2:55 vs. 5:17 min) versus standard imaging, without compromising overall image quality or pathology detection [75]. Similarly, another clinical series reported a ~61% reduction in exam time with DL reconstruction, with the accelerated MRI showing significantly less noise and artifacts, higher sharpness, and even greater diagnostic confidence than the standard scans [76,77]. In short, these DL-MRI techniques allow faster spine scans (reducing patient discomfort and motion blur) while preserving or boosting image clarity.

DL is also used to generate or enhance MRI sequences that would otherwise require extra scan time. For instance, a synthetic STIR (Short Tau Inversion Recovery) image can be generated by a neural network from routine T1/T2 images. One study showed that synthetic STIR reduced the dedicated STIR acquisition by ~3 min, yet yielded significantly higher signal-to-noise ratio (SNR) and contrast-to-noise ratio (CNR) than a standard STIR; radiologists found the synthetic STIR images diagnostically interchangeable with the real STIR [78]. Likewise, in cervical spine imaging, applying DL reconstruction to zero-echo-time (ZTE) MRI, a CT-like sequence, greatly improved bone detail. Blinded readers rated the DL-enhanced ZTE images as having “superior image quality and bone visualization” compared to conventional ZTE, making it easier to evaluate osseous stenosis [79]. These examples show that AI can synthesize or enhance MRI contrasts, speeding protocols and improving visualization of both soft tissue and bone, without losing diagnostic information [78,79].

In spine CT, AI-powered reconstruction mainly targets noise reduction and dose savings. Modern scanners already use iterative reconstruction, but DL reconstruction (e.g., GE’s TrueFidelity or ClariCT.AI) can push quality further. In one 2025 clinical study, lumbar CT scans were processed with a DL denoising algorithm, ClariCT.AI, and compared to standard filtered back-projection [80]. The DL-reconstructed CT showed a much higher sensitivity for detecting disk herniations (60% vs. 44%) and higher specificity for stenosis than standard CT; radiologists rated the DL-reconstructed CT images as having superior image quality and diagnostic confidence [80]. In other words, the AI denoising made subtle soft tissue findings on CT more conspicuous, improving clinical performance. Phantom studies echo these benefits: an experiment using GE Healthcare’s DL image reconstruction model, TrueFidelity, showed that deep learning reconstruction dramatically reduces noise and boosts spatial resolution and lesion detectability compared to hybrid iterative methods [81]. This implies that for a given image quality, radiation dose could be substantially lowered using DL image reconstruction techniques.

AI can also fuse modalities to enhance spine evaluation. A striking example is MRI-based synthetic CT (sCT) of the spine. In a multicenter study of acute cervical spine trauma, an AI algorithm generated CT-like bone images from routine MRI. The sCT images detected 97.3% of fractures (sensitivity) and showed near-perfect agreement with actual CT in measuring vertebral heights and alignment [82]. The authors concluded that sCT is a “promising, radiation-free” approach with accuracy comparable to CT [82]. In practice, this means a patient could undergo one MRI exam and obtain both soft tissue and CT-like bone information, reducing the need for a separate CT.

Together, these studies illustrate the real-world clinical value of AI reconstructions. DL-enhanced MRI protocols allow substantially shorter spine exams (often 40–60% faster) while maintaining or improving image quality [76,77]. DL-denoised CT scans enable a lower radiation dose with equal or better disease detection [80,81]. Synthesized sequences like STIR or ZTE-DL maintain full diagnostic content with far less scan time [81]. Additionally, multimodal tools like sCT combine the strengths of MRI and CT in one step [82]. Many of these AI reconstructions are now integrated into commercial systems (for example, FDA-cleared software like SubtleMR uses DL back-projection networks trained on millions of MRI images) so that radiology departments can apply them in routine spine imaging [76].

### 3.3. AI’s Ability to Enhance Workflow

AI is increasingly being applied to optimize clinical workflow in spinal imaging. One key frontier is scanning triage, where AI algorithms automatically prioritize studies that show critical or urgent findings, ensuring that radiologists review these cases first and reducing delays in time-sensitive diagnoses [83,84].

AI triage tools are increasingly deployed to flag urgent spinal pathologies and streamline radiology workflow. For example, Aidoc’s FDA-cleared cervical-spine fracture algorithm automatically analyzes trauma CT scans and prioritizes patients with potential fractures by sending them to the top of the radiologist’s patient list [13]. In practice this means the radiologist is alerted to subtle C-spine fractures within minutes.

This quick alert helps reduce diagnostic time; researchers found a 16 min (46%) reduction in time to diagnosis for positive cases of cervical spine fractures when using Aidoc compared to traditional methods [63]. These spine-imaging triage systems that automatically detect acute findings (e.g., cord compression or vertebral fractures) and push those studies up the queue can substantially improve radiologist efficiency. Faster turnaround for urgent cases not only speeds diagnosis but can improve outcomes: the American College of Radiology notes that AI detection of acute cord compression could “reduce turnaround time and improve quality of care,” potentially averting paralysis or other deficits [85].

In sum, AI triage in spine imaging ensures that critical findings are recognized and reported more rapidly. Systems like Aidoc’s C-spine algorithm platform illustrate how deep learning can flag urgent pathology and automatically notify clinicians. This process improves overall workflow by focusing radiologist attention on high-priority cases first, reducing the risk of missed injuries, and shortening diagnostic delays. Early clinical results suggest that these tools can significantly cut report wait times and length of stay for patients. Moreover, opportunistic AI screening (e.g., algorithms that detect vertebral fragility fractures on routine CT) can identify otherwise undetected spine injuries in thousands of patients. By integrating AI alerts into the PACS and reporting system, radiology departments can achieve faster turnaround, more consistent detection of acute spine findings, and more efficient communication with treating teams. However, recent external validation studies have highlighted important limitations, particularly in detecting chronic fractures and subtle pathological changes amid varying fracture characteristics and imaging quality [86,87]. This is a cautionary tale to developers and hospital administration alike: all parties must ensure continuous model refinement and, if needed, failure mode analysis to optimize clinical performance.

Large language models (LLMs) and Natural Language Processing-driven (NLP-driven) tools are beginning to automate the creation of radiology reports from spine imaging data. Many current systems combine image analysis (e.g., segmentation, detection, measurements) with templated language output. For example, SmartSoft’s FDA-cleared CoLumbo software Version 3 analyzes lumbar spine MRIs, identifying and measuring vertebral structures and pathologies, and then creates a detailed draft report with the findings that clinicians can edit [88]. Similarly, RemedyLogic’s Radiology AI platform creates concise summaries on its findings after identifying abnormalities and incidental findings [89]. In practice, these systems pre-populate structured report templates with key observations (e.g., levels of stenosis, disk degeneration) and draft language, which the radiologist can then review and finalize. These reports in turn should help improve workflow and save radiologists’ time. Studies have shown that LLM-assisted reporting can significantly reduce reporting time compared to conventional methods, with one study demonstrating a reduction from 8.95 to 6.76 min per report using summary-based workflows and LLM-generated templates, without compromising report quality [90]. Another study found that multimodal LLMs using minimal audio input reduced reporting time and corrections compared to conventional speech recognition workflows, while maintaining or improving report quality [91] LLMs also support multilingual and personalized reporting, further streamlining the process [92].

Beyond rule-based reporting, generative AI is also being introduced into radiology workflows. For instance, Nuance/Microsoft’s PowerScribe Smart Impression uses an LLM to automatically draft the impression section of a report from the imaging findings that are highly rated for scientific terminology, coherence, and comprehensiveness; these reports either matched or exceeded human performance in clarity and completeness [93]. In a similar spirit, one study fine-tuned an 8-billion-parameter Llama-3 model on thousands of MRI and CT reports and found that the LLM could generate clinically accurate descriptions for new imaging exams [94]. These proof-of-concept systems illustrate how an LLM can ingest structured findings and output clinically sensible narrative text, thereby lightening the radiologist’s workload and improving report uniformity.

Importantly, integrating AI outputs into the reporting workflow has proven efficiency benefits. For example, an “AI-to-structured-report” pipeline was developed for chest X-rays, in which AI findings automatically populate a structured report template. Reports generated with this pipeline were completed in significantly less time (mean ~67 s) than traditional free-text reports (~86 s) and were rated as higher quality [95]. Translating this to spine imaging, a similar workflow could automatically fill out a spine MRI or CT report with measurement values and descriptions (e.g., disk height, neural foramina size), vastly reducing administrative burden. In practice, these tools not only accelerate reporting but also standardize reports. Consistent language and structured output ensure that referring clinicians (e.g., surgeons, pain specialists, etc.) receive clear, comparable information across patients. By reducing repetitive transcription and variability, AI-generated preliminary reports help radiologists focus on complex interpretation and ensure that key findings are communicated efficiently and uniformly to the clinical team.

## 4. Surgical Planning and Robotic-Assisted Interventions

### 4.1. Advanced Preoperative Planning and Simulation

Artificial intelligence (AI) has transformed preoperative planning in spinal surgery, shifting the paradigm from generalized heuristics toward patient-specific, data-driven optimization. Traditional imaging review and surgeon experience remain foundational, but modern AI-powered platforms now extract detailed biomechanical and anatomical features from CT and MRI to build 3D reconstructions tailored to each patient [16]. These reconstructions are not static visual aids; they increasingly integrate predictive analytics, offering simulations of surgical trajectories, guide implant selection, and anticipated biomechanical stresses.

One significant development is the incorporation of finite element analysis (FEA) into AI-driven platforms. By simulating load-bearing mechanics under various conditions, FEA helps forecast implant longevity, risk of hardware failure, and the distribution of spinal stresses post-fusion [96]. Such modeling has direct clinical relevance, especially in osteoporotic patients or those undergoing multilevel fusions where the interaction between native bone and implants can be unpredictable.

Robotic platforms are embedding these tools into their workflows. The Mazor X Stealth Edition integrates predictive planning with robotic navigation, enabling surgeons to simulate screw placement in virtual environments before transferring the plan intraoperatively [17]. Similarly, the ExcelsiusGPS provides real-time feedback during execution, linking preoperative CT-based models with intraoperative adjustments that account for cortical thickness and bone mineral density. These features are particularly valuable in deformity or revision surgeries, where conventional anatomic landmarks are unreliable [17,19,97].

Peer-reviewed validation studies highlight the accuracy of such systems. Jia et al. demonstrated that an AI-driven planning algorithm designed screws that were not only larger and longer than those chosen manually, but also safer: 85.1% of AI-suggested screws achieved a Grade A accuracy (i.e., no cortical breach) compared to 64.9% in freehand techniques [98]. Similarly, a DL-based planning system using the nnU-Net architecture successfully segmented spinal anatomy and recommended trajectories on par with human experts, although its robustness across highly variable pathologies remains an open question [99].

Imaging innovation is extending planning into radiation-conscious domains. A study by Levi Chazen et al. showed that deep learning-reconstructed 3D MRI could mimic CT for pedicle screw planning, achieving nearly identical geometric accuracy [100]. This approach could significantly reduce patient exposure to ionizing radiation, especially in young or revision-prone populations.

The next frontier lies in adaptive, intelligent planning frameworks. The SafeRPlan model exemplifies this evolution, applying deep reinforcement learning (DRL) to refine screw trajectories intraoperatively. Importantly, it integrates safety filters to mitigate errors, representing a step toward autonomous yet surgeon-supervised planning systems [101]. Such “learning-in-action” methodologies mark a shift away from static preoperative designs toward continuously adaptive surgical strategies.

### 4.2. Robotic-Assisted Surgical Execution

Robotics in spine surgery is evolving from navigational assistance toward true AI-driven co-surgeons. Early systems like ROSA Spine or da Vinci SP functioned primarily as stabilizers for surgeon-guided trajectories, but modern platforms increasingly incorporate AI for dynamic adjustment and real-time control [102].

The VELYS™ Active Robotic-Assisted System, developed by DePuy Synthes with eCential Robotics, exemplifies this shift. FDA-cleared for use across cervical, thoracolumbar, and sacroiliac fusions, VELYS employs active robotics with independent navigation that recalibrates in response to intraoperative imaging and even subtle patient movement [102,103]. This millimeter-level adaptability is particularly critical in revision and deformity surgeries where unexpected tissue shifts or hardware interference often derail traditional navigation.

Medtronic has also advanced robotic intelligence by linking its Mazor platform with real-time analytics. The system integrates intraoperative CT, fluoroscopy, and MRI, dynamically updating trajectories when deviations occur [104]. This “closed-loop” design reduces cumulative error, a limitation often cited in earlier-generation systems. Likewise, the eCential Robotics Spine Suite consolidates planning, navigation, and execution into one environment, enabling continuous recalibration across modalities and surgical phases [105,106,107].

Importantly, the trend is toward self-improving systems. Cloud-based federated learning allows robotic platforms to assimilate global datasets, tracking outcomes across institutions, while maintaining patient privacy [108]. This collective intelligence could eventually normalize performance across centers, reducing variability that currently depends heavily on surgeon skill and institutional resources.

Early experimental work suggests where this is heading: DRL-driven robotic frameworks that autonomously adjust trajectories in real time. These models learn to adapt to intraoperative conditions such as tissue deformation, with built-in safeguards to ensure error containment [101]. While still investigational, their translation into clinical robotics could herald a new era of semi-autonomous intraoperative adaptation.

### 4.3. Integration with Advanced Navigation and Guidance Systems

Navigation technologies remain the backbone of precision in spine surgery, but AI is fundamentally reshaping how guidance is achieved. Brainlab Curve Navigation, for example, now couples intraoperative CT and fluoroscopy with AI-based registration to accelerate setup and improve accuracy in pedicle screw placement [109]. This reduces not only operative time but also the radiation burden from repeated imaging.

Stryker’s NAV3i expands this model through automatic landmark recognition, which simplifies registration in anatomically complex cases such as scoliosis [27]. Medtronic’s StealthStation S8 similarly enhances adaptability by continuously correcting navigation drift using AI-driven error modeling [28]. These advances address one of the most persistent problems in spine navigation: progressive misalignment during surgery.

Zimmer Biomet’s ROSA ONE Spine and NuVasive’s Pulse platforms add further layers with AI-enabled augmented reality (AR) overlays. These overlays visualize planned screw paths directly in the surgical field, while feedback systems dynamically warn surgeons of deviation from optimal parameters [110,111,112]. Such human–machine synergy enables surgeons to anticipate complications in real time rather than respond reactively.

Emerging techniques now bypass traditional markers altogether. A deep neural network was recently used to segment the lumbar spine directly from RGB-D camera input, enabling AR-guided navigation with registration errors averaging ~1.2 mm and 100% pedicle screw accuracy in ex vivo testing [113]. Similarly, a modular hybrid system achieved ~1.1 mm accuracy using as few as three C-arm images per screw, underscoring the feasibility of combining AI-guided efficiency with reduced radiation exposure [114].

Together, these approaches illustrate the convergence of AI, robotics, and AR/VR into fully integrated surgical ecosystems, where each component strengthens the accuracy and safety of the others.

### 4.4. Functional Outcome Prediction and Treatment Optimization

Beyond intraoperative execution, AI is increasingly applied to predicting recovery and optimizing long-term outcomes. This is particularly relevant in spine surgery, where radiographic success does not always correlate with patient satisfaction or functional improvement.

Recent work with Graph Neural Networks (GNNs), GAN, and Transformer models demonstrates the potential of integrating diverse datasets, ranging from imaging biomarkers to psychosocial indices, to predict complications, pain persistence, or quality-of-life outcomes [115,116,117,118]. These models extend beyond statistical regression, uncovering nonlinear patterns invisible to conventional analytics.

**Figure 3:**
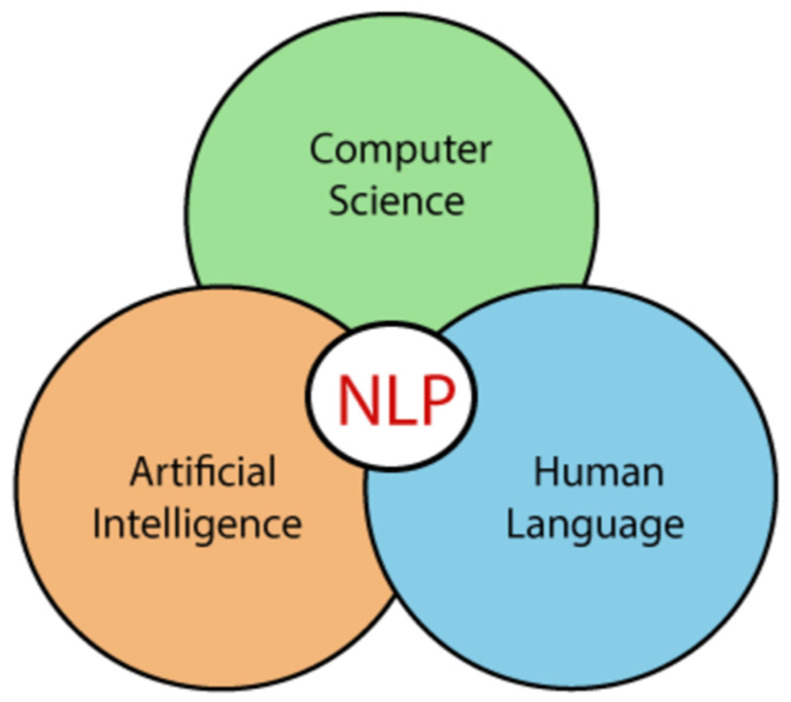
Diagram showing the hierarchy of Natural Language Processing. The depicted text is ineligible for copyright and therefore in the public domain because it is not a “literary work” or other protected type in sense of the local copyright law [122].

NLP represents another frontier. By mining clinical notes, postoperative journals, or patient-reported text, NLP algorithms can quantify subtle psychological factors such as anxiety, resilience, or depressive sentiment that heavily influence recovery (Figure 3) [119,120,121]. Such insights could individualize rehabilitation strategies in ways radiographic assessment cannot.

Wearable technology provides the data backbone for these predictive frameworks. Devices such as ActiGraph GT9X and WHOOP Strap 4.0 generate continuous physiological, activity, and sleep data [123,124]. When analyzed through AI models, these datasets create dynamic recovery profiles, guiding interventions like physiotherapy intensity or pain management titration. Edge AI and federated learning approaches allow such analyses while preserving patient privacy, enabling multicenter datasets to inform global predictive models [125].

Early pilot studies suggest that integrating intraoperative robotic performance metrics (e.g., screw placement accuracy, time to completion) with postoperative wearable-derived physiology may produce holistic predictions of recovery trajectories. Such models could ultimately drive adaptive, individualized rehabilitation regimens, optimizing both functional outcomes and patient satisfaction.

### 4.5. Cost-Effectiveness Analysis

Despite these technical advances, the sustainability of AI in spine surgery depends on its economic justification. Health systems operating under value-based care frameworks increasingly demand demonstration that technology improves outcomes relative to costs [126]. Yet, spine surgery lacks disease-specific quality-of-life (QOL) instruments that adequately capture its unique blend of functional, neurological, and psychosocial outcomes [74,127,128].

Cost-effectiveness studies of navigation and robotics generally report improved accuracy, shorter operating times, and reduced complication rates. However, high acquisition and maintenance costs, coupled with steep training curves, remain major barriers to adoption [129]. These findings underscore the need for comprehensive cost models that incorporate both direct (equipment, training, OR time) and indirect (rehabilitation, reoperation avoidance) variables.

Future frameworks may need to incorporate behavioral economics and complexity theory, assessing not just cost per procedure but how surgeon learning, institutional adoption, and interdisciplinary workflows evolve in response to AI integration [9,129]. Coupling these models with predictive outcome analytics could allow reimbursement structures that tie financial incentives directly to demonstrated patient-centered improvements.

## 5. Genomic Applications and Precision Medicine

### 5.1. Genome-Wide Association Studies in Spine Surgery Risk Assessment

Large-scale genome-wide association studies (GWAS) leveraging national biobanks (e.g., UK Biobank, FinnGen) are beginning to clarify the heritable architecture of spinal disease and perioperative risk. In surgically managed adult spinal deformity (ASD), a study of 540 patients identified 21 single-nucleotide polymorphisms (SNPs) associated with surgical risk, with the LDB2 variant (rs12913832) showing the most robust signal and implicating ectodermal differentiation pathways in spinal morphogenesis and repair [21,130,131,132,133,134]. Parallel meta-analyses for lumbar disk herniation (LDH) across three biobank cohorts reported 41 novel loci, implicating genes central to inflammatory signaling (IL6R), extracellular matrix composition (COL11A1), and Wnt/β-catenin regulation (DKK1)—pathways that mechanistically influence disk integrity and biomechanical behavior. For lumbar spinal stenosis (LSS), replicated associations near GFPT1 and AAK1 point to roles for glycosylation and synaptic vesicle trafficking in modulating neural vulnerability to compressive forces [132,133,134,135,136].

Translating these associations into clinical utility requires aggregation and contextualization. Polygenic risk scores (PRS) that combine genome-wide variant weights with clinical covariates (body-mass index, radiographic severity, comorbidity indices) now permit probabilistic stratification of patients for adverse outcomes, examples include heightened predicted risk for pseudarthrosis tied to SMAD3-related signaling and elevated reoperation probability in certain genetic strata [137,138]. Importantly, integrating GWAS signals with phenotypic features using machine learning models, where genomic data are fused with radiomics and clinician notes parsed by large language models, has improved prediction of early neurological deterioration and postoperative sepsis in ASD cohorts, enabling earlier, preemptive resource allocation [139]. Clinically actionable findings are emerging: carriers of CHST3 variants, which associate with degenerative disk phenotypes, may be prioritized for intensified rehabilitation or considered for targeted biologic strategies (e.g., matrix metalloproteinase inhibitors in MMP2-linked stenotic disease). These represent early but concrete instances of genomics informing individualized perioperative plans.

Beyond risk assessment, the field is exploring interventional genomics. Ongoing translational efforts and early-phase trials investigate gene-editing (e.g., CRISPR-Cas9) and small-molecule modulation of GWAS-implicated targets such as NFU1 in stenosis and GSDMC in inflammatory disk degeneration, highlighting a therapeutic horizon where genotype guides targeted molecular therapy in conjunction with surgical management [140].

### 5.2. Pharmacogenomics and Personalized Pain Management

Pharmacogenomics is increasingly relevant to postoperative analgesia and medication safety in spine surgery. Allelic variation in cytochrome P450 enzymes—principally CYP2D6 and CYP3A4—substantially influences opioid pharmacokinetics and clinical response: CYP2D6 poor metabolizers risk inadequate conversion of prodrugs such as codeine, whereas ultra-rapid metabolizers face overdose risk from active metabolites, a nuance critical for perioperative prescribing [141]. Clinical decision support systems (e.g., PharmCAT) and consensus resources from CPIC translate genotype (including OPRM1 rs1799971) into EHR-integrated prescribing guidance, enabling clinicians to tailor opioid selection or choose alternatives such as tapentadol or non-opioid adjuncts (e.g., duloxetine for COMT Val158Met carriers) [142,143,144]. Similarly, polymorphisms in inflammatory mediators, IL6 rs1800795 and TNF-α rs1800629, have been associated with differential NSAID efficacy and adverse-event profiles, informing safer anti-inflammatory choices in at-risk patients [145]. For bone healing, genetic variation in BMP2 (rs235768) and the vitamin D receptor (VDR rs731236) have been used to triage patients who may benefit from anabolic agents such as teriparatide to reduce nonunion risk following fusion [146,147]. Commercial pharmacogenomic panels (e.g., OneOme RightMed) are broadening their gene coverage and are increasingly incorporated into pre- and postoperative workflows to mitigate adverse events and optimize analgesic efficacy. Together with the shift toward non-addictive agents (gabapentin for neuropathic pain; SNRIs for centralized pain syndromes), these genomic tools support individualized, safer multimodal pain regimens in the post-opioid-epidemic era [148,149].

### 5.3. Multi-Omics Analysis

Integrative multi-omics platforms operationalize complex molecular datasets to delineate pathobiology and predict therapeutic response. Cloud-enabled pipelines (Seven Bridges, DNAnexus) now permit the harmonized analysis of genomics (GWAS/PheWAS), proteomics (e.g., Olink Target 96 inflammation assays), metabolomics (Metabolon HD4), and single-cell transcriptomics (10× Genomics) to map dysregulated pathways in spinal disease [150]. Proteomic profiling by proximity–extension assays has repeatedly identified elevations in IL-6 and COMP in degenerative disk disease, while high-throughput proteomics (Somalogic SomaScan 7K) has associated increased MMP-3 expression with subsequent pseudarthrosis, suggesting candidate biomarkers for failed fusion risk [151]. Machine learning frameworks such as DeepOmics now integrate these modalities, linking specific genomic variants (e.g., COL1A1 mutations) to downstream collagen dysregulation and ACAN variants to proteoglycan depletion and disk desiccation, thereby predicting which molecular phenotypes may respond to particular biologic or regenerative interventions [22]. As these multiomic signatures are validated longitudinally and embedded into clinical decision pipelines, they will enable precision stratification of patients for targeted biologics, optimized surgical timing, and personalized rehabilitation regimens.

## 6. Clinical Decision Support and Documentation Systems

### 6.1. Ambient Clinical Intelligence and Documentation Automation

Ambient clinical intelligence (ACI) platforms have substantially reconfigured documentation workflows in contemporary practice. Systems such as Nuance Dragon Ambient eXperience (DAX) and Suki AI combine advanced speech recognition with natural language processing to generate clinical notes directly from physician–patient conversations [23]. These platforms can diarize encounters, identify salient clinical findings, and structure documentation according to specialty-specific templates and guideline-driven frameworks. When integrated with electronic health records (EHRs), ACI tools enable scribes, nurses, and physician assistants to document more efficiently and with greater consistency, reducing duplication of effort across the care team.

Specialty-focused charting solutions further extend these capabilities. For example, Ambience Healthcare provides co-pilot functionality for pre-charting, real-time scribing, and automated post-visit summaries [48]. Its connectivity with major EHR vendors supports bidirectional data exchange and richer secondary analysis of clinical information [48]. Similarly, DeepScribe and Notable Health illustrate how deep learning-based systems can be tailored for orthopedics and spine care. These platforms are trained on large, domain-specific datasets so that their output reflects spine anatomy and pathology in addition to surface linguistic features; this allows for more clinically relevant error detection and correction than grammar-only dictation tools.

Across implementations, the principal reported benefit is a reduction in after-hours documentation and associated clerical burden, which can improve clinician work–life balance and reduce the risk of burnout. Importantly, these technologies are positioned as augmentative rather than replacement tools: clinicians retain supervisory control, reviewing and validating algorithm-generated content. This human–AI collaboration functions as a check-and-balance that both streamlines documentation and mitigates charting errors, while preserving clinician accountability and the integrity of the medical record.

### 6.2. Clinical Decision Support Systems

AI-enabled clinical decision support systems (CDSS) deliver real-time, evidence-based guidance to assist diagnosis, therapeutic selection, and management optimization in spine care. IBM Watson for Clinical Decision Support exemplifies the marriage of cognitive computing and clinical expertise, mining large bodies of medical literature together with patient-level data to produce tailored treatment recommendations [14,152]. Such systems can synthesize complex presentations to generate differential diagnoses, suggest treatment options, and estimate prognoses that reflect both current best evidence and individual patient characteristics.

Critical to their utility is the ability to integrate heterogeneous data streams (e.g., imaging features, demographics, comorbidities, and operative variables) to perform comprehensive risk stratification and outcome prediction. Continuous learning mechanisms permit these systems to incorporate new research findings and real-world outcomes, progressively refining their recommendations. Moreover, real-time analytics deliver dynamic risk updates as a patient’s status evolves or as intraoperative information becomes available, enabling proactive adjustments to management plans that aim to optimize clinical outcomes.

## 7. Current Challenges, Limitations, and Implementation Barriers

### 7.1. Technical and Algorithmic Limitations

The adoption of novel AI technologies into routine spine care remains constrained by several interrelated technical and algorithmic limitations. Prominent examples illustrate limited generalizability: systems developed for cervical spine fracture detection (e.g., Aidoc) and joint assessment (e.g., Zebra Medical Vision’s HealthVCF) have demonstrated performance drops when applied to imaging produced under different protocols or scanner vendors. Variations in slice thickness, field of view, and contrast administration commonly degrade model performance when these algorithms encounter unfamiliar datasets. In practice, complete harmonization across major vendors (e.g., GE, Siemens, Philips) is unrealistic, and algorithm robustness must therefore be addressed through design and deployment strategies rather than idealized standardization [15,153,154].

Hardware-related artifacts further complicate reliable performance. Metallic implants, frequent in revision spine surgery, produce signal distortions that have led to segmentation failures in systems such as SpineNet when processing MRI scans with titanium hardware [155]. These fragilities raise both patient-safety and economic concerns, because models require ongoing surveillance, periodic retraining, and software maintenance to remain clinically useful, increasing implementation and lifecycle costs.

Algorithmic limitations also arise from the intrinsic heterogeneity and rarity of certain pathologies. SpineNet and similar tools exhibit weaker performance in grading disk degeneration and quantifying spondylolisthesis for less common conditions; examples include sacral chordomas, which present atypical anatomy, histopathology, and metastatic patterns that are poorly represented in training sets [156,157,158]. Although enlarging and diversifying training datasets can mitigate some failures, it is neither practical nor feasible to expect any system to achieve perfect sensitivity and specificity across every rare presentation.

Dataset bias and representativeness are additional, persistent problems. Publicly available collections (e.g., CTSpine1K) and many institutional repositories disproportionately reflect urban, academic practice populations, leaving rural and underserved patient presentations underrepresented. Geographic, linguistic, and practice-pattern variations therefore create sampling biases that limit external validity. Retrospective curation further compounds the issue through selection effects. Consequently, no training corpus can be entirely comprehensive or error-free, an unavoidable constraint that must be acknowledged during model evaluation and clinical deployment.

Longitudinal assessment poses yet another technical challenge. Many current models are cross-sectional by design, inhibiting consistent tracking of disease progression across pre- and postoperative intervals. This limitation is particularly consequential for practices without access to high-performance computing resources. Smaller private groups and many community hospitals may be unable to bear the USD 500,000–USD 1 million capital costs associated with real-time processing infrastructure, a disparity that persists alongside the closure of over 140 U.S. hospitals since 2010 [159,160,161]. Finally, limited model interpretability, the so-called “black box” problem, can obscure decision rationale, impairing clinician trust and complicating the translation of algorithmic outputs into patient-centered care [162].

### 7.2. Regulatory and Validation Challenges

Regulatory pathways and validation requirements introduce additional barriers. The FDA’s evolving framework struggles to accommodate continuous learning systems, such as those embedded in platforms like Mazor X Stealth Edition, where post-market performance may change as algorithms adapt. Worryingly, a 2022 analysis found that 226 of 521 FDA-cleared AI devices lacked peer-reviewed clinical validation, raising questions about the evidence base supporting many deployed tools and the potential risks to patient safety [51].

Robust, multi-institutional validation, necessary to demonstrate generalizability across heterogeneous care settings, is logistically and financially demanding. Collaborative validation efforts that coordinate datasets across rural and urban hospitals have been estimated to cost on the order of USD 250,000 annually, limiting participation by resource-constrained centers and slowing generation of high-quality external evidence [163]. The ALIGNMENT study provides a cautionary precedent: despite guideline dissemination, uptake of short-course radiotherapy did not increase substantially, illustrating how dissemination alone cannot substitute for practical validation and institution-specific implementation work [164].

Physician skepticism compounds these challenges. Concerns about workflow disruption, loss of autonomy, and uncertain reimbursement influence adoption decisions, small practices are particularly sensitive to economic pressures, and recent Medicare payment reductions for spine procedures (2024) have further constrained capital investments such as robotic platforms (e.g., ExcelsiusGPS) [165]. Regulatory and documentation burdens, reporting requirements that can add an estimated 10–15 administrative hours per week, further disincentivize adoption in understaffed clinics. Collectively, these forces create a conservative adoption environment where only well-validated, cost-effective, and workflow-friendly solutions are likely to gain traction.

### 7.3. Clinical Integration and Workflow Challenges

Finally, integrating AI into the clinical ecosystem challenges established workflows and cultural norms. Intergenerational differences shape expectations: some senior clinicians view tools like automated scribes (e.g., DeepScribe) as encroachments on clinical autonomy, while newer clinicians expect seamless EHR integration and interoperability to reduce clerical burden [166]. Patient acceptance is equally critical and increasingly fragile; high-profile healthcare data breaches in 2024 have heightened privacy concerns and made patients more cautious about the use of AI/ML tools, especially those that perform sentiment analysis or rely on extensive, linked datasets. Addressing these integration barriers requires not only technical robustness and regulatory clarity but also transparent governance, clear communication about data stewardship, and implementation strategies co-designed with clinicians and patients to preserve trust and clinical utility.

### 7.4. Data Quality, Generalization, and Statistical Stability

Despite promising diagnostic accuracy in controlled research settings, AI-driven spine imaging tools remain fundamentally constrained by data quality and representativeness. Convolutional neural networks (CNNs) implicitly assume that training and clinical images share the same statistical properties; any distribution shift (e.g., different scanner vendors, acquisition protocols, or patient populations) can markedly degrade performance [167,168]. Studies consistently report that models trained at one center often underperform on external data, with accuracy drops when applied to images from unfamiliar sites or devices [167,168]. In short, the “garbage in, garbage out” principle holds: CNNs learn superficial statistical cues in the training set and therefore rely critically on high-quality, well-curated data [24,167]. If data are noisy, biased, or limited in scope, model outputs become unreliable; for instance, mislabeled cases or subtle imaging artifacts in the training set will propagate directly to model errors, since CNNs are not inherently robust to annotation errors [24,167]. Additionally, large-scale datasets can increase statistical power but also introduce distinct risks when data provenance, sampling biases, or measurement semantics are not explicitly handled. Roccetti et al. describe a useful cautionary example: training an RNN on ≈15 million water-meter readings from >1 million meters initially produced non-positive results until the authors defined a clear “semantics of validity” and curated a representative subset, at which point model accuracy rose from roughly 60% to the 80–90% range [169]. This work illustrates a practical paradox: more data does not automatically resolve bias, and indiscriminate upscaling can amplify noise or confounders rather than improve real-world performance. Consequently, we emphasize the need for (i) careful data provenance reporting, (ii) explicit criteria for inclusion/exclusion and semantic validation of records, and (iii) external validation across heterogeneous cohorts before asserting clinical or operational generalizability [169].

Typical medical datasets are often small, imbalanced, and collected under narrow conditions. High-quality labels require expert effort and are expensive, so many available datasets contain annotation errors, ambiguity, or limited case diversity [24,170]. Rare spine pathologies and uncommon patient demographics may be underrepresented, causing CNNs to overfit common patterns and miss atypical cases. This problem has been highlighted by Alizadehsani et al. and others, who show that data scarcity and class imbalance significantly impair CNN robustness and generalizability [167,170]. Ensuring statistical stability and that training data truly reflect real-world variability is therefore a major obstacle. Practical remedies include continuous external validation, assembling multicenter training cohorts, and applying domain-adaptation techniques to maintain performance across diverse clinical settings [168,170]. Without such rigorous data curation and ongoing model refinement, AI systems risk failing in non-ideal conditions and should be applied with caution.

Training data should be expertly annotated to cover the full spectrum of disease presentations and patient demographics to ensure reliability and clinical relevance. In reality, datasets vary widely in annotation quality and scope: multi-site collections can suffer from inconsistent protocols and labeling conventions, and retrospective curation often favors easily obtainable cases (e.g., urban hospitals or clear-cut pathology), thereby omitting rare presentations or underrepresented populations [171]. This dataset bias, when training data do not fully reflect the target patient population, produces models that perform well on familiar cases but degrade on new scenarios [171]. Recent reviews therefore emphasize the need to standardize data collection and annotation across institutions and to explicitly document dataset provenance (e.g., imaging vendor and patient mix) so users can assess generalizability [171].

Beyond data composition, statistical rigor in model evaluation is essential for stability and trust. Models trained on small or narrow datasets are prone to overfitting, and a number of systematic reviews have found that many published AI studies rely solely on cross-validation with small samples and omit sample-size calculations, a significant methodological shortcoming [171]. Without proper power analysis or independent test sets, reported accuracies may be inflated or highly variable. Developers should therefore compute confidence intervals for performance metrics and, whenever possible, reserve a held-out test set or perform external split-validation. Larger, multi-institutional datasets are also needed: empirical evidence suggests that increasing sample size often yields lower, but more realistic, accuracy estimates, implying that some earlier small-sample reports were over-optimistic [171]. Reporting only a single metric (e.g., a solitary AUC) without confidence bounds obscures the potential range of true performance and can undermine clinical trust.

Finally, AI systems must remain statistically stable over time. After deployment, data distributions commonly drift because of changes in equipment, protocols, or patient populations; even well-calibrated models can misbehave on shifted inputs. Responsible implementations therefore include ongoing data monitoring and drift detection (e.g., checking input histograms or outcome rates), with retraining or recalibration triggered as needed. Regulatory guidance is beginning to address these concerns: for adaptive or continuous learning systems, an Algorithm Change Protocol is advised to specify retraining criteria and requisite validation steps after any update [24]. In sum, ensuring high data quality (i.e., comprehensive, consistently annotated training sets), applying rigorous statistical validation, and explicitly quantifying uncertainty (e.g., with confidence intervals and external testing) are cornerstones of robust, generalizable AI in healthcare.

### 7.5. Bias, Fairness, and Subgroup Performance

AI tools can unintentionally perpetuate or worsen healthcare disparities when training data reflects historical biases. Experts caution that deployment must assess not just overall accuracy but performance across demographic subgroups [172]. For example, recent studies have found that chest X-ray classifiers tend to systematically underdiagnose disease in Black patients [172]. Deep learning models have even been shown to infer sensitive attributes (race, gender, age) from imaging data, using them as “shortcuts” [172], learning correlations that exist in the data but have no true causal basis. If unaddressed, such biases can lead to unequal care (e.g., delayed treatment for one group), defeating the equitable intent of AI.

To uncover and mitigate these biases, subgroup performance must be reported explicitly. Recent reporting guidelines (i.e., TRIPOD-AI) now require that model evaluations include metrics with confidence intervals for each key subgroup (e.g., race, sex, age, or other clinically relevant categories) [173]. In practice, this means a developer should present, say, the sensitivity and specificity separately for men vs. women or for different racial groups. This can reveal hidden failures: for instance, a model with 90% sensitivity overall might have only 75% sensitivity in a minority subgroup. Encouragingly, TRIPOD-AI explicitly embeds fairness in its checklist with the “issues of fairness” in the interpretation section and advises the use of subgroup plots or heterogeneity analyses [173]. By contrast, many older studies omitted such details, making it impossible to know if an algorithm is safe for all populations. Demanding transparent subgroup reporting is thus a practical step toward fairness in spine imaging and beyond.

When biases are identified, steps should be taken to mitigate them. These steps might include rebalancing training data (e.g., adding more examples from underrepresented groups) or incorporating fairness-aware algorithms that penalize disparate error rates. Importantly, even good fairness performance in the training context is not guaranteed to hold after deployment: models must also be tested for bias under distribution shifts [172]. Ultimately, ensuring equitable AI in medicine requires both (a) rigorous measurement of subgroup outcomes and (b) thoughtful adjustment of models and workflows to correct any unfairness.

### 7.6. External Validation, Robustness, and Failure-Mode Testing

Demonstrating an AI model’s robustness requires testing beyond its own training environment. Numerous systematic reviews have found that radiology AI algorithms suffer performance drops when tested on external data. In one review, fully 81% of published external validations showed a decline in accuracy on new datasets, with about a quarter showing large (≥0.10) drops in AUC [174]. This drop is strong evidence that models often “overfit” to the specifics of one site. For this reason, current best practice is to perform multi-center external validation before deployment. Ideally, developers should evaluate their model on data from several hospitals or vendors that were not used in training, to ensure it generalizes across scanners and patient populations. Indeed, experts urge a shift from single-center studies to multi-center trials and prospective evaluations [29,56]. Such validation might include, for example, scanning new MR images from a partner institution to see if the AI’s performance holds steady.

Robustness to edge cases and data shifts is also critical. AI systems should be explicitly stress-tested with challenging cases. A concrete illustration of the pitfalls of scale comes from Roccetti et al. (2019), who found that training on a very large, uncurated time-series initially produced poor results until the dataset’s “semantics of validity” and inclusion criteria were defined and a representative subset curated, after which performance improved substantially [169]. This example highlights that stress-testing should include checks for dataset validity and semantic consistency in addition to the usual perturbation/failure-mode analyses. Domain experts recommend failure-mode analysis by identifying scenarios where the model fails and why as part of validation [29]. For instance, specialized testing of a spine fracture detection model revealed that false negatives clustered in patients with severe degenerative changes or metallic hardware, common in spine surgery [30]. These were “stress tests” the model had not seen. By systematically generating or collecting such cases, teams can quantify how performance degrades. Similarly, models should be checked for sensitivity to common perturbations (e.g., motion artifact, different slice thicknesses, etc.), since real-world images are often noisier than textbook examples. Reporting robustness measures (e.g., worst-case error or uncertainty estimates on outlier inputs) provides critical context for safe use.

A further layer of validation comes from shadow deployments. Before entrusting AI with patient care, many institutions run it in parallel: the model receives real imaging data and makes predictions, but clinicians do not act on them. This “shadow mode” allows observation of the AI’s real-time performance and failure modes without any risk to patients [31]. During this phase, developers can monitor for calibration drift (i.e., changes in accuracy over time) and gather user feedback. Regulatory experts suggest that even low-risk automation tools undergo shadow evaluation, while higher-risk systems (like autonomous decision-making) ultimately need prospective trials [31,32]. In practice, this staged rollout (i.e., silent shadowing to supervised testing to live use) has been a common feature of successful AI integrations, as it catches issues that retrospective tests may miss.

### 7.7. The Gap Between Promising Research and Clinical Reality

Despite promising results on curated datasets, AI models have inherent limitations that temper their clinical utility. First, most AI algorithms do not truly “understand” disease the way humans do; they learn statistical patterns, which may not hold outside the training context [171]. In medical imaging, this is manifested as dataset bias: available data often do not fully represent the clinical spectrum of patients. For example, one review notes that common practice is to train on convenience samples (e.g., cases from academic centers) that only partially reflect real patient populations [171]. In consequence, a model that performs well on a benchmark can “fail catastrophically” when faced with a new hospital’s images [171]. In fact, a systematic review of COVID-19 imaging AI found none of 62 studies had demonstrable readiness for clinical use [35], illustrating how overfit solutions on limited data may not translate to practice. These findings underscore that AI is not a panacea; models should always be seen as supporting, not replacing, clinical judgment, especially in novel scenarios. More broadly, scale cannot substitute for semantic validation: studies found that increasing dataset size without explicit semantics of validity can amplify noise and confounders rather than improve real-world performance [171]. Therefore, practical deployment strategies should pair dataset expansion with rigorous semantic curation and transparent inclusion/exclusion rules.

Second, many technical challenges remain. Deep learning models are often “black boxes” with limited interpretability. This opacity can hinder trust and obscure failure modes; currently there is no guarantee that a given model will behave sensibly on a rare case or under adversarial perturbations. Relatedly, most AI studies focus on single-timepoint classification and do not model patient longitudinally or causally; they lack an understanding of progression over time. There is also the issue of calibration: AI confidence scores can be poorly calibrated, giving a misleading sense of certainty. Handling domain shifts and adversarial examples, intentional or not, remains an active research problem. In short, current models do not self-correct when facing truly new challenges.

Finally, practical deployment limitations cannot be ignored. Regulatory pathways for AI are still evolving, and most models enter the clinic with limited evidence: few have randomized trial validation or real-world outcome studies. Even with validation, implementation requires robust IT infrastructure, privacy safeguards, and user training, which are obstacles that many hospitals struggle with. Economic and workflow factors (e.g., cost of hardware, integration into PACS, clinician workload) often become rate-limiting. In the spine imaging domain in particular, the relatively small volume of cases and the diversity of pathologies mean that algorithmic improvements have to overcome a high bar of cost and complexity. In sum, AI promises much but also has real constraints: its outputs must be interpreted in the context of clinical judgment, sound data practice, and ongoing surveillance. Only through acknowledging and addressing these limitations can safe, effective AI tools be realized.

## 8. Discussion

The body of evidence synthesized in this review demonstrates that artificial intelligence (AI) and machine learning (ML) are no longer exploratory curiosities in spine care; they are operational technologies that touch imaging pipelines, preoperative planning, intraoperative navigation, postoperative monitoring, and even early translational genomics [3,9,10,11,13,16,17,19,43,63,70,71,83,84,85,96,97]. Across these domains, the recurring theme is one of tremendous potential tempered by real-world limits: algorithmic brittleness when exposed to heterogeneous data, infrastructural and economic barriers to equitable deployment, gaps in external validation and regulatory oversight, and non-trivial cultural and workflow frictions among clinicians, patients, and institutions. Below we synthesize the clinical implications of the evidence, examine mechanistic reasons for persistent performance gaps, and propose a pragmatic translational roadmap that aligns technical priorities with regulatory and health-system realities.

From a practical standpoint, AI has already demonstrated clinically meaningful benefits where tasks are structured, repetitive, and well-defined. Automated fracture and acute-pathology triage systems shorten time-to-diagnosis in emergent settings [29,30,31,32,56], opportunistic screening algorithms identify otherwise-missed fragility fractures [63], and segmentation/morphometric pipelines markedly reduce measurement variability for alignment metrics such as Cobb angle and sagittal vertical axis (SVA) [13,44,63,83,84,85]. In preoperative planning and robotics, predictive modeling and finite-element-assisted planning improve the precision and reproducibility of screw trajectories and implant selection [88,89,90,91,92,93,94,95]. Meanwhile, emerging multimodal prognostic models that integrate imaging, wearable data, and genomic signals offer a new avenue to personalize perioperative risk-reduction strategies, rehabilitation intensity, and pharmacologic plans [3,9,10,11,43,70,71]. Collectively, these capabilities point to a hybrid clinical model in which AI augments clinician judgment, automates low-value work, and provides earlier, finer-grained signals to inform decision-making.

However, these practical benefits are often undermined by persistent performance gaps, particularly when models are deployed beyond their development environments. Recent reports showing markedly higher diagnostic accuracies than older studies likely reflect multiple converging factors, not solely algorithmic breakthroughs. Larger, better-curated training corpora, improved network architectures and transfer-learning methods, and more sophisticated image-reconstruction pipelines have raised retrospective test metrics. At the same time, reporting practices (e.g., use of internal test sets, selective case sampling, and the occasional presence of data leakage) and publication bias toward positive results can inflate apparent performance. Crucially, prospective and external evaluations frequently reveal lower, more clinically realistic performance (see, e.g., external evaluations of triage algorithms), underscoring that headline accuracy numbers should be interpreted in the context of dataset provenance, validation methodology, and deployment environment. For deployment decisions and regulatory assessment, emphasis should be placed on multi-center prospective validation, calibration reporting, and transparent failure-mode analysis.

Additionally, several interlocking technical realities explain the recurring drop in performance when models leave their development environments. First, domain shift is pervasive: scanner vendors, slice thickness, acquisition protocols, contrast timing, and local reporting conventions create distributional differences that degrade generalizability [15,153,154]. Second, the presence of metallic hardware or unusual anatomy produces imaging artifacts and edge cases that were underrepresented in training sets, generating catastrophic failure modes in segmentation and measurement algorithms [155]. Third, many current models are trained on retrospective, single-center cohorts that overrepresent tertiary-care populations; the result is geographic and socio-economic selection bias that erodes external validity [156,157,158]. Fourth, cross-sectional model designs hamper longitudinal monitoring; a model trained for single-timepoint classification struggles to track progression or the effects of serial interventions [159,160,161]. Finally, opaque model explanations, the “black box” problem, hamper clinician trust and obstruct effective failure-mode analysis [162]. These technical deficits are compounded by human and organizational factors: clinicians resist workflows that feel unstable or require substantial retooling, administrators balk at high upfront capital costs for compute and devices [163], and patients worry about privacy and data misuse [164,165,166].

Beyond these technical and human challenges, current regulatory pathways were not designed for adaptive, continuously learning systems deployed across thousands of variable clinical contexts. Analyses show that many AI devices entered the clinical domain with limited peer-reviewed validation and that post-market performance monitoring is inconsistent [20,51]. For safe, equitable scaling, regulators and clinical stakeholders must converge on practical expectations for premarket evidence (external, multi-institutional validation), transparent reporting, and robust post-market surveillance. A key step in this modernization is for the FDA to mandate several guidance upgrades for spine AI. A risk-stratified evidence model is needed: low-risk tools such as automated measurements should demonstrate analytic validity across multiple sites and undergo a supervised “shadow mode” deployment before approval. Moderate-risk systems, like diagnostic triage and surgical planning assistants, should be required to prove external validity across diverse patient groups, include calibration analyses by demographic and technical subgroups, and report at least one prospective clinical impact study. High-risk tools, particularly autonomous or closed-loop robotic systems, must meet the most stringent standards, such as randomized or controlled prospective trials paired with robust post-market surveillance. Beyond these evidentiary thresholds, guidance should mandate disclosure of dataset provenance, including geographic distribution, vendor diversity, and patient demographics, so that reviewers can gauge generalizability. For adaptive systems, an Algorithm Change Protocol should be required, specifying retraining conditions, validation steps after updates, rollback procedures, and clinician/end-user notifications when performance shifts occur. To safeguard clinical use, vendors should also provide regular real-world performance dashboards, including sensitivity, specificity, and calibration metrics broken down by demographic categories, and be subject to mandatory adverse-event reporting when misclassification leads to harm or near-miss events.

While governance is critical, evidence also suggests that requiring a technology to be clinically useful is not the same as designing it to be deployable. Successful implementations share several features: native interoperability with EHRs (FHIR/HL7 compliance), minimal workflow disruption (e.g., integration that populates structured report fields rather than forcing transcription changes), clinician-facing explainability (clear, concise model outputs with uncertainty estimates), and a staged roll-out with shadow mode, supervised use, and then live deployment [14,23,48,152]. Change management also matters: clinician champions, multidisciplinary AI governance committees, and clear metrics of clinical utility (turnaround time, diagnostic yield, patient-centered outcomes) accelerate adoption. Economic models that account for total cost of ownership, including retraining, maintenance, and regulatory reporting, better predict institutional willingness to adopt [163].

To address the issue of equitable access, the promise of AI magnifies existing inequities if models are trained and validated on narrow cohorts. Federated learning and privacy-preserving analytics provide technical pathways to broaden training datasets while minimizing raw data movement, but these must be paired with deliberate inclusion strategies (such as incentives for rural/underserved centers to participate in consortia) and prospective audits of model calibration across demographic strata [47]. Patient trust hinges on transparent data governance, explicit consent for secondary uses, auditable data lineage, and rigorous cybersecurity measures; the high-profile breaches of recent years have demonstrably eroded public confidence and must guide any implementation strategy [164,165,166].

Ultimately, to move the field forward, research must shift from single-center optimization to multi-center external validation and prospective impact trials. Priority areas include robustness engineering (domain-adaptation methods, uncertainty quantification, and explicit failure-mode detection for edge cases such as metal hardware and severe deformity); longitudinal modeling frameworks for consistent tracking of disease state across serial imaging and clinic visits; explainability and human–AI interaction studies that produce clinically meaningful visualizations of model reasoning; federated and distributed learning initiatives to improve representativeness without compromising privacy; cost-effectiveness and outcomes research that couples clinical outcomes, quality-of-life instruments, and economic impact; and open benchmark datasets with reporting checklists (e.g., TRIPOD-AI, CONSORT-AI adaptations) [159,160,161].

The FDA should also emphasize transparency and explainability: models should disclose interpretability methods, provide standardized uncertainty metrics, and include clinician-facing output templates that clearly distinguish prediction, confidence, and suggested action. Interoperability standards (e.g., HL7/FHIR compliance, standardized labeling) must be enforced to reduce integration costs and ensure comparability across platforms. Finally, equity and cybersecurity must be central. Systems should report subgroup-level performance, incentivize validation that includes underrepresented populations, and adhere to rigorous encryption, penetration testing, and vendor transparency standards. Through these measures, FDA guidance can both protect patients and accelerate innovation in AI for spine care [51,163].

## 9. Conclusions

AI and ML technologies have reached a point of clinical maturity in specific, high-value tasks within spine care: emergent triage, standardized morphometrics, surgical planning assistance, and nascent prognostic modeling [3,9,10,11,13,16,17,19,43,63,70,71,83,84,85,96,97]. These technologies promise measurable improvements in diagnostic speed, reproducibility, and personalization of perioperative care. However, the translation of algorithmic promise into broad, safe clinical benefit is not automatic. Persistent technical fragilities, including domain shift, hardware artifacts, limited longitudinal modeling, and underrepresentation of diverse populations, combined with unequal access to infrastructure, insufficient external validation, and regulatory gaps, constrain equitable deployment [15,51,153,154,155,156,157,158,159,162].

To move from demonstration to durable clinical impact, the field requires aligned action on three fronts. First, technical innovation must prioritize robustness, explainability, and longitudinal fidelity, not just peak accuracy on curated datasets. Second, implementation science must make AI a practical clinical collaborator through seamless interoperability, clinician-centered interfaces, staged rollouts, and careful change management [14,23,48,152,163]. Third, regulators and health systems must modernize governance frameworks with risk-proportionate evidentiary requirements, transparent dataset reporting, algorithm-change protocols, mandatory post-market surveillance, and equity safeguards [51].

Concretely, an upgraded FDA approach, one that combines stratified premarket standards, adaptive system governance, enforced external validation, ongoing real-world performance auditing, and interoperability mandates, will both protect patients and accelerate clinically meaningful adoption. Parallel investments in federated learning, subsidized computational infrastructure for resource-limited centers, and incentives for multi-center prospective validation studies will prevent a two-tiered system in which AI-enhanced care is only accessible in well-resourced institutions [159,160,161].

In sum, AI is poised to become an integral, augmentative element of contemporary spine care. Yet the pathway to safe, equitable, and effective scale depends as much on governance, workflow design, and economic strategy as it does on algorithmic advances. A coordinated effort among developers, clinicians, regulators, payers, and patients, guided by transparent validation, robust monitoring, and explicit equity goals, will be essential to realize AI’s promise while minimizing foreseeable harm.

## Figures and Tables

**Table 1:** Overview of AI applications in spine surgery and diagnostic workflows. This table categorizes current AI-driven technologies across various domains of spine care, including diagnostic imaging, surgical planning, intraoperative navigation, functional outcome prediction, and economic modeling. Each entry outlines the specific AI tools or systems in use, their validation status, clinical benefits, and known limitations. The table is designed to offer clinicians, researchers, and health policymakers a concise reference for evaluating the maturity, clinical utility, and future development needs of spine-focused AI systems.

AI/ML Application Area	Key Tools/Systems	Validation Status	Clinical Benefit	Limitations
Fracture Detection and Classification	Zebra HealthJOINT [24,25], Aidoc Cervical Spine AI [1,26,27,28]	FDA Approved; Real-world validation	Reduced under-detection; Improved triage accuracy	Limited chronic fracture detection; Sensitivity varies
Spinal Segmentation and Grading	SpineNetV2 [3,29,30,31], Multimodal Segmentation Platforms	External validation across modalities	Automated grading of stenosis, disk degeneration	Performance may vary across demographics
Morphometric Analysis	CobbAngle Pro Version 1 [32,33,34], Yeh et al. Ensemble Model [35]	Validated vs. clinical experts	Reduced measurement error; Field-applicable	Dependence on image quality
Ultrasound-based Imaging	UGBNet [36], Attention-Unet [7]	Peer-reviewed feasibility studies	Segmentation of low-contrast images	Noise sensitivity in complex anatomy
Muscle Quality Quantification	CTSpine1K [37,38], TrinetX [38]	Open-source annotated datasets	Cross-sectional muscle area and fat infiltration	Need for standardized protocols
Preoperative Planning	Mazor X [3,5,39,40], ExcelsiusGPS [5,39,41]	Clinical integration with robotic systems	Optimized screw trajectory, virtual planning	Variable accuracy in deformed anatomy
Robotic Execution	VELYS [10,20], ROSA Spine [5,42], Mazor Robotics [10]	FDA-cleared, commercial use	Real-time trajectory correction; Error reduction	Cost and infrastructure requirements
Navigation and Guidance	Brainlab Curve [43], Medtronic StealthStation [10]	Integrated AI + imaging validation	Adaptive navigation; Improved pedicle accuracy	Setup complexity; Intraoperative variability
Outcome Prediction	GNNs, Transformers, Sentiment NLP [44,45,46,47]	Ongoing studies; Cross-disciplinary use	Predict functional recovery, mental health monitoring	Integration of heterogeneous data types
Cost-Effectiveness and QOL Modeling	Dynamic Simulations, Complexity Economics	Emerging models; Not yet widespread	Forecasting long-term impact; Behavioral insights	Lack of spine-specific QOL instruments

**Table 2:** Technical limitations and implementation barriers to clinical integration. This table delineates key challenges impeding the widespread adoption of AI in spine diagnostics and surgery, categorized across imaging variability, algorithmic brittleness, dataset bias, explainability deficits, regulatory complexity, infrastructure costs, and clinical integration issues. Each barrier is described in detail, along with its technical considerations, and paired with its practical clinical or operational consequences. The table aims to guide researchers, developers, and healthcare administrators in identifying systemic vulnerabilities and prioritizing translational improvements necessary for safe and scalable AI deployment in spinal healthcare.

Category	Barrier	Technical Detail	Clinical/Operational Consequence
Imaging and Model Generalizability	Cross-Vendor Imaging Variability	Heterogeneity in scanner vendor output (e.g., GE vs. Siemens vs. Philips) causes domain shift in AI models; non-uniform slice thickness and FOV distort CNN feature extraction layers.	Decreased classification precision for compression fractures; high false-negative rates in under-standardized imaging environments.
Hardware-Induced Artifacts	Metallic Implant Interference	Titanium-induced susceptibility artifacts in T1/T2 MRI sequences disrupt segmentation accuracy in deep neural networks like SpineNet and V-Net variants.	Invalidated predictions in post-fusion patients; potential for underestimation of central canal and foraminal compromise.
Pathological Heterogeneity	Low Representation of Rare Tumors	Model sensitivity drops when exposed to rare presentations (e.g., sacral chordomas, extradural myxopapillary ependymomas) due to weak class priors and minimal edge-case training data.	False negatives in tumor surveillance; unreliable outputs for oncological follow-up assessments.
Training Data Bias	Geographic and Socioeconomic Overfitting	Training sets skewed toward tertiary care centers cause latent space misalignment for rural/underserved demographics; manifests as calibration drift in diagnostic AI systems.	Inaccurate prioritization in triage algorithms; potential exacerbation of healthcare disparities.
Model Explainability	Opacity in Neural Attribution Maps	Lack of saliency map interpretability or explainable AI (XAI) frameworks in real-time decision support; attention-based models still fall short in spine-specific pathologies.	Limited clinician trust in AI output; inability to validate or refute system recommendations during multidisciplinary rounds.
Infrastructure and Cost	High-Cost HPC Requirements	Inference latency optimization via GPU clusters (e.g., NVIDIA A100) requires capital investment exceeding $500k; suboptimal throughput without federated inference pipelines.	Barriers to adoption in rural and small private clinics; delayed implementation in mid-tier health systems.
Regulatory and Legal Complexity	Validation of Continuous Learning Systems	Regulatory frameworks not equipped for post-deployment model drift; challenge in validating self-updating AI modules under FDA’s Good Machine Learning Practice (GMLP) guidelines.	Post-market liability ambiguity; disincentivizes procurement by risk-averse hospital administrators.
Workflow and Physician Engagement	Non-Interoperability with Legacy EHRs	Lack of native HL7/FHIR compliance in AI tools (e.g., DeepScribe); interface incompatibility leads to fragmented data workflows and redundancy in documentation.	Cognitive overload and duplication of work; rejection by high-volume providers.
Patient-Centric Barriers	Privacy Anxiety from Data Breaches	2024 cyberattack exposure of biometric and imaging datasets undermines patient confidence in AI-driven diagnostics; hesitancy persists even with federated learning protocols.	Consent withdrawal and decreased utilization of AI-assisted care; limits scalability of patient-facing applications.

## Appendix A. Technical Background on CNNs

### Appendix A.1. Rationale and Overview

At the core of many of these applications are Convolutional Neural Networks (CNNs), a class of deep learning models specially designed to mimic the human visual cortex in how they process and analyze visual data. They can ingest large image datasets and automatically extract spatial features from raw pixels to hierarchically learn subtle patterns of disease [24].

### Appendix A.2. Architecture, Layers and Feature Hierarchy

CNNs are built from repeating blocks of convolutional and pooling layers, often followed by one or more fully connected, dense layers [24,25]. To start, each convolutional layer applies a set of trainable filters across the image, producing feature maps that highlight where certain patterns (e.g., an edge, texture, shape) occur. Pooling layers then downsample these feature maps, reducing spatial resolution while preserving the strongest signals. This pooling confers spatial invariance: small shifts in the image do not drastically change the pooled characteristics [25]. By alternating convolution and pooling layers, the network builds a hierarchy of features; early layers capture simple structures (e.g., lines or edges) while deeper layers combine them into complex shapes or pathology-specific patterns [24,52]. In practical terms, low-level convolutional filters might respond to simple edges or blobs, whereas higher-level filters in deeper layers respond to organs or lesions. Finally, one or more fully connected layers aggregate these features to produce a prediction [24,25]. In summary, CNNs automatically learn to detect and synthesize the relevant spatial features in an image, mimicking the brain’s hierarchical visual processing [24].

### Appendix A.3. Training, Tasks, and Outputs

CNN models are trained on large, annotated imaging datasets (e.g., MRI, CT or X-ray scans labeled by experts) split into training and test sets (e.g., an 80:20 training–test split) [53]. During training, the network’s filter weights are iteratively adjusted via backpropagation and gradient descent to minimize the difference between the network’s predictions and the ground-truth labels [24]. For classification tasks, the final output of the CNN is a categorical label (e.g., “tumor present” vs. “absent,” or disease severity grading); the network learns to output class probabilities. In localization or detection tasks, the network produces spatial outputs. For example, object-detection CNNs (e.g., Faster R-CNN) regress bounding-box coordinates to surround each abnormality, while segmentation CNNs (e.g., U-Net) output pixel-wise masks delineating the lesion [26,54]. Some networks also produce heatmaps that highlight the image regions most influential for the decision [55]. In practice, CNNs excel at both pixel-level tasks (e.g., identifying the exact region of a tumor) and image-level tasks (e.g., classifying an entire scan), often achieving high sensitivity and specificity once well-trained. Their fully connected layers effectively translate the hierarchical features into final predictions, enabling breakthroughs in diagnostic accuracy and patient care [25,52].
